# Genomic Variants Associated With Oocyte and Embryo Production in Dairy Gir Cattle

**DOI:** 10.1002/age.70098

**Published:** 2026-04-12

**Authors:** Renata de Fatima Bretanha Rocha, Haniel Cedraz de Oliveira, Marta Fonseca Martins, Marco Antônio Machado, João Claudio do Carmo Panetto, Marcos Vinícius Barbosa da Silva, Simone Eliza Facioni Guimarães

**Affiliations:** ^1^ Department of Animal Science Universidade Federal de Viçosa Viçosa MG Brazil; ^2^ Embrapa Dairy Cattle Research Center Juiz de Fora MG Brazil

**Keywords:** dairy cattle, mutations, reproductive traits, variant call file

## Abstract

The use of in vitro fertilization protocols associated with genomic selection increases genetic gain in dairy cattle. The aims of this study were to uncover genetic variants in a specific interval on BTA7 and to investigate their effects on the number of oocytes and embryos in dairy Gir. Previous research uncovered a region on chromosome 7 (BTA7) associated with oocyte and embryo production. In the current study, genomic variants were investigated in this region for 12 sires with positive Predicted Transmission Ability for milk production, which are widely used in dairy herds to produce daughters for oocyte and embryo production. Several variants were identified and four variants were classified as lead SNPs. ANOVA was carried out for the daughters' genomic estimated breeding value (GEBV) and phenotypes according to the genotype of the bulls. Two lead SNPs were single‐nucleotide substitutions and both resulted in premature stop codons in *XRCC4* and *HAPLN1* genes. A deletion of five thymines in an intergenic region occurred in the third lead SNP. The fourth lead SNP involves the deletion of one nucleotide in the *EDIL3* gene, causing a frameshift. All genotype groups were significantly different for GEBV and some for phenotype. In conclusion, the mutant alleles of each lead SNP were associated with the production of oocytes and embryos in the dairy Gir, leading to a decrease (rs518509552, rs438544900), an increase (rs450555472) when homozygous or a decrease (rs470818992) when heterozygous. Research is underway to investigate the association of such variants with dairy production and environmental resilience traits.

**Abbreviations**
EMBR: number of in vitro produced embryos
TO: total number of oocytes
VO: number of viable oocytes

## 1 Introduction

Recently, the remarkable rise in the adoption of cattle assisted reproductive technologies in Brazil has been globally recognized, underscored by an increase in in vitro embryo production (IVEP) from approximately 12 500 in 2000 to over 300 000 annually after 2010 (Sartori et al. 2016). According to the International Embryo Technology Society (IETS 2025), Brazil is the second largest IVEP producer, surpassed only by the USA. In 2023, over one million straws of embryo transfer (ET) were sold in Brazil. However, due to the growing market for low‐cost embryos, not all ET are registered by the breeder associations, indicating that the numbers of IVEP and ET in Brazil are likely higher than official estimates (Viana 2024). In Brazilian dairy cattle, the increase in IVEP started with the availability of sex‐sorted semen and the increase in the percentage of frozen–thawed embryos relative to fresh ones, which has been associated with the adoption of in vitro technologies such as in vitro fertilization replacing superovulation, along with greater oocyte yield of zebu breeds (Viana et al. 2017). The joint use of reproductive technologies and genomic selection can enhance genetic gain by providing higher quantity and quality oocytes from females with high Predicted Transmission Ability (PTA) and by selecting young animals, which reduces the generation interval and accelerates genetic progress (Sirard 2018; Hansen 2023).

With advances in reproductive biotechnologies in livestock, there has been an increase in studies exploring the genomic aspects of oocyte and embryo production in cattle (Cornelissen et al. 2017; Watanabe et al. 2017; Ferré et al. 2023). These studies are common in zebu breeds, such as the dairy Gir (Lacerda et al. 2020; Vizoná et al. 2020; Rocha et al. 2022), which show high potential for milk production in tropical climates, resistance to heat and parasites, respond better to ovulation protocols, and yield more oocytes than taurine breeds (Feltes et al. 2022). Nevertheless, there is a lack of genomic variants explored in the literature for fertility traits, especially for *Bos indicus* breeds. Currently, the OMIA (Online Mendelian Inheritance in Animals) catalogue of genes and variants (Nicholas et al. 2025) includes 733 phenotype records for taurine and 58 records for zebu cattle, among which include a small percentage of studies in reproductive traits mainly in male subfertility, fetal death, fecundity, haplotypes with homozygous deficiency (lethal alleles), and short tail in sperm. Therefore, more studies to identify variants associated with fertility traits in zebu cattle are needed.

Genomic selection for reproductive traits is of great importance in dairy farming, as these traits generally are polygenic and have low heritability, allowing for genetic gains earlier than with traditional selection (Tade and Melesse 2024). Although there are few known mutations affecting bovine reproduction, EMBRAPA Dairy cattle publishes in bull summaries the genotypes of animals for some lethal traits, such as the uridine monophosphate synthase deficiency, which is caused by a homozygous embryonic lethal allele, complex vertebral malformation, which causes severe deformities in the spine and joints of fetuses leading to pregnancy losses, and bovine brachyspina, which causes skeletal malformation retarding fetal growth and resulting in abortions or stillbirths (Panetto et al. 2025a). Information on bull genotypes helps in identifying carriers, selecting the most suitable animals, and avoiding risky matings, which is useful to farmers and breeders.

In a recent study, a genomic region on *Bos taurus* autosome (BTA) 7, located between nucleotides 82974837 and 83997563 was identified in Gir sire families and associated with the number of total oocytes, viable oocytes, and embryos (Rocha et al. 2024a). Furthermore, a previous study examining the parent‐of‐origin of alleles confirmed the influence of the paternal lineage on the total oocyte production in Gir females (Rocha et al. 2024b). However, few studies have identified high‐impact mutations that influence the number of oocytes collected and embryos produced in livestock farming. The current study aimed to fill this gap, uncovering genetic variants in this BTA7 interval and investigate their effects on the number of oocytes and embryos in the dairy Gir families under investigation. The hypothesis for our analysis is that mutations affecting oocyte and embryo production in dairy Gir cattle are found in the region BTA7:82974837 −83997563. This research revealed four mutations in the bovine genome that are associated with the number of oocytes and embryos in cattle and the GEBV of Gir breed animals for these traits.

## 2 Materials and Methods

### 2.1 Population and Data

For a comprehensive understanding of the current work, details of the previous research can be found in the Open Access article by Rocha et al. (2024a). In brief, a family analysis was performed using the daughter design approach (Weller et al. 1990). For that, 15 genotyped Gir sires with more than 20 daughters each were selected from a pedigree file containing 4679 Gir animals. These sires had positive PTA for milk production, and thus they are widely used in Brazil to produce Gir and Girolando cattle. Genomic information for the sires and their daughters was provided by the National Dairy Gir Breeding Program, and phenotypic information for the daughters was provided by 5 farms in Minas Gerais State, including the number of viable oocytes (VO), the number of total oocytes (TO), and the number of in vitro produced embryos (EMBR). The search for genomic regions associated with these traits was performed through GWAS analyses among and within families. Ultimately, a genomic region on BTA7, between nucleotides 82 974 837 and 83 997 563 ( *Bos taurus* genome assembly ARS‐UCD1.2), was identified as the most commonly inherited region among the families.

### 2.2 Variant Check

For the current study, a variant calling format (VCF) file containing substitutions or insertions and deletions (indels) variants for all 29 autosomal chromosomes of 43 Gir sires was checked. From that file, 12 sires from the previous research were recovered. Subsequently, variants in the region BTA7: 82974837–83997563 were extracted using BCFtools (Danecek et al. 2021) for further analyses. The extracted VCF file of the BTA7 region was analyzed using reference bovine genome assembly ARS‐UCD2.0 using the online tool EnsemblVEP (McLaren et al. 2016) to determine the consequences of the variants. Variants with a high impact classification in EnsemblVEP were considered for further analysis as lead SNPs and were visually reviewed and validated in the Integrative Genomics Viewer (IGV) (Robinson et al. 2023).

The VCF file was phased using Beagle software (version 5.5, Browning et al. 2021) and then transformed into the PLINK program format (version 1.9; Chang et al. 2015) to calculate linkage disequilibrium (LD) between each lead SNP and other variants. Next, 20 upstream and 20 downstream variants from each lead SNP's position with an *r* ^2^ > 0.8 were selected to build the haplotypes using vcfR package in R (Knaus and Grünwald 2017).

### 2.3 Genomic Estimated Breeding Values (GEBV)

A genotype file with 56 092 animals genotyped using SNP arrays of different densities, including Illumina BovineSNP50 BeadChip v2 (50 K), Illumina BovineHD BeadChip (777 K), GGP Indicus (34 K), ZChip (30 K), and GGP Indicus (50 K), was imputed for the 777 K panel. The file contained 420583 SNPs before quality control. GEBVs were obtained with the BLUPF90+ software family. The default values of this program were used for genomic quality control, resulting in 55439 genotyped animals and 389194 SNPs from bovine autosomal chromosomes. The phenotype dataset included 102836 information for VO, TO and EMBR from 11649 Gir donors, of which, 8546 had genotype information. All these animals are part of the genomic evaluation of the National Dairy Gir Breeding Program carried out by EMBRAPA Dairy Cattle, located in Juiz de Fora, Minas Gerais (Panetto et al. 2025b). The animals originated from various lineages within the breed, representing a significant sample of the Brazilian Dairy Gir population. Because the dataset was compiled from multiple breeding companies that performed in vitro fertilization in Gir cattle across different farms, data quality control was carried out following the procedures described by Rocha et al. (2022). The pedigree file for the donors included 198770 animals, covering 21 generations.

The traits were transformed using a logarithmic scale, ln (*X* + 1) to obtain a normal distribution of the residuals. GEBV was calculated using the BLUPF90+ software family (Lourenco et al. 2022), considering the following model:

y=Xβ+Za+Wp+e

where ***y***, ***β***, ***a***, ***p***, and ***e*** are the vectors of observations; fixed effects; additive genetic random effects; permanent environment random effects and residual effects, respectively; ***X***, ***Z***, and ***W*** are the incidence matrices of fixed, additive genetic, and permanent environment, respectively.

For VO and TO, 2483 contemporary groups (CG) were available, which was considered as a fixed effect. CG included 304 farms, 9 companies that performed Ovum Pick Up (OPU), OPU year (2005–2025) and season (1: January–March, 2: April–June, 3: July–September, and 4: October–December). For EMBR, the fixed effects of CG, bull (*n* = 962) used in the insemination process, and bull breed (Gir, *n* = 25399; Holstein, *n* = 76067; Jersey, *n* = 381) were considered. Donor's age in days (linear and quadratic components) was included as covariates for all traits. OPU interval (linear component) was included as a covariate for VO and TO. The additive genetic, permanent environment and residual effects were assumed to be random.

### 2.4 Statistical Analyses

Daughters were grouped according to their sire's genotype for each of the lead SNPs, which were considered as high impact variants according to EnsemblVEP. The normal distribution was verified with the “nortest” package (Gross and Ligges 2015). Analyses of variance (ANOVA) and *t*‐tests were performed using the R software (version 4.4.3; R Core Team 2025) for GEBV and phenotypes of each trait, considering genotype groups (G). To evaluate the traits, the proposed model was:

Yij=μ+G+e

where *Yij* is the GEBV/phenotype of the *i*‐th observation from the *j*‐th genetic group, *μ* is the overall mean and e is the random error associated with the observation. Statistical significance was considered when *p*‐value < 0.05 and the Student's *t*‐test was used to compare averages.

### 2.5 Haplotype Blocks

For each sire, a file with their daughters' genotype was extracted. The “hsphase” package (Ferdosi and Gondro 2025) was used to build graphs within the predetermined region and visualize recombination events. The haplotype blocks for the graphs were based on the genotypes of half‐sisters within each family for the whole chromosome seven and for the BTA7: 82974837–83997563 interval.

## 3 Results

Descriptive statistics of the data used is available in Table 1. OPU interval ranged from 83.67 to 160 days, and the donors' age ranged from one to 17 years. The number of viable oocytes obtained from this 20 years OPU dataset was around 1 million; for total oocytes, it was almost 2 million, and for embryos, it was over 400 thousand, with an average of 15 for VO, 20 for TO, and 4 for EMBR.

**TABLE 1:** Descriptive statistics^a^ on structures, donors' age, Ovum Pick Up (OPU) interval, number of OPU per donor, and number of animals per CG.

	Number of observations	Total	Mean ± SD	MIN	MAX	CV
Viable oocytes	102 832	1 596 351	15.52 ± 12.10	0	268	77.95
Total oocytes	99 399	1 995 405	20.07 ± 14.39	0	320	71.67
Embryos	102 804	406 206	3.95 ± 4.27	0	81	108.09
Age (years)	—	—	5.95 ± 3.10	1	17	52.14
Ovum Pick Up interval (days)	—	—	83.67 ± 160.94	0	4163	192.35
Number of OPU per donor	—	—	17.96 ± 14.10	2	85	78.49
Number of animals per CG	—	—	91.55 ± 82.64	3	352	90.27

^a^ CG, contemporary group; CV, coefficient of variation; DP, standard deviation; MAX, maximum; MIN, minimum (0 is the minimum in the Ovum Pick Up interval when two samples were collected from the same cow on the same day and fertilized with semen from different bulls); Total, sum of total structures in the dataset; Variables are not transformed to a logarithmic scale.

The variants within the studied region on BTA7 included 7341 indels and 43 335 SNPs. From these, 6365 SNPs and 1086 indels were located in non‐coding regions. The number of SNPs and indels found on genic regions were 8851 and 1581 for *EDIL3*, 1335 and 318 for *HAPLN1*, 5885 and 1135 for *VCAN*, and 20 899 and 3221 for *XRCC4*, respectively. The impact of variants in EnsemblVEP was classified as modifier, low, moderate, and high impact (Table S1). A high impact classification indicates that the variant has a disruptive effect on the protein sequence, such as protein truncation, loss of function, or triggering nonsense‐mediated decay. In our study, four variants in the BTA7: 82974837–83997563 interval were classified as high impact and were considered lead SNPs for further analyses. The sire genotypes of four lead SNPs and the number of daughters per sire are displayed in Table 2 for all 12 sires.

**TABLE 2:** Description^a^ of the number of daughters, lead SNP genotype, genomic estimated breeding value (GEBV), and accuracy (*r*) for each trait per dairy Gir sire.

Sires	Number of phenotyped daughters	Number of phenotypes	Lead SNP 1	Lead SNP 2	Lead SNP 3	Lead SNP 4	Total oocytes		Viable oocytes		Embryos	
			rs518509552	rs438544900	rs450555472	rs470818992	GEBV	*r*	GEBV	*r*	GEBV	*r*
s1	2049	20 589	0/1	0/1	1/1	0/0	−0.14	0.99	−0.13	0.99	−0.17	0.99
s2	1565	16 648	0/0	0/0	1/1	0/1	0.09	0.99	0.08	0.99	0.17	0.99
s3	940	7403	0/0	0/0	1/1	1/1	−0.38	0.99	−0.35	0.99	−0.18	0.99
s4	590	5474	0/0	0/0	1/1	1/1	0.38	0.99	0.30	0.99	0.20	0.99
s5	540	4432	0/0	0/1	0/1	1/1	−0.20	0.99	−0.18	0.99	−0.31	0.99
s6	481	4754	0/0	0/1	0/1	1/1	−0.36	0.99	−0.28	0.99	−0.41	0.99
s7	318	3206	0/0	0/1	1/1	1/1	−0.21	0.99	−0.19	0.99	−0.43	0.99
s8	212	1561	0/0	0/0	1/1	0/1	−0.73	0.99	−0.72	0.99	−0.53	0.99
s9	179	1478	0/1	0/0	1/1	1/1	−0.58	0.99	−0.60	0.99	−0.37	0.99
s10	147	1342	0/0	0/0	1/1	0/1	0.02	0.98	0.09	0.98	−0.03	0.98
s11	68	695	0/0	0/0	1/1	0/1	−0.32	0.98	−0.30	0.98	−0.37	0.98
s12	41	423	0/0	0/0	1/1	0/1	−0.35	0.97	−0.25	0.97	−0.19	0.97

^a^ Lead SNP: variant with high impact according to EnsemblVEP in position 7:82.974.837–83.997.563 ( *Bos taurus* Genome Assembly ARS‐UCD1.2); 0/0: homozygous for wild allele; 0/1: heterozygous; 1/1: homozygous for mutant allele.

Lead SNPs 1 and 2 were single‐nucleotide substitutions, and both resulted in stop codons within the protein coding sequence, causing protein truncation. Lead SNP 1 (7:83178164) is located in the *XRCC4* gene (ENSBTAT00000131234.1, exon 8/8), where a cytosine (C) was replaced by a guanine (G). Lead SNP 2 (7:83600471) is located in the *HAPLN1* gene (ENSBTAT00000098839.1, exon 2/2), where a cytosine (C) was replaced by a thymine (T). Lead SNP 3 (7:83814505) showed two alternative alleles in the original VCF file, where alternative allele 1 is the deletion of five sequential thymines and alternative allele 2 is the deletion of four thymines. For the 12 sires in this research, only the alternative allele 1 was present. Although it was the only variant that occurred in an intergenic region, it is located in a long non‐coding ribonucleic acid region (lncRNA), resulting in a splice variant at an intron region of this lncRNA. Lead SNP 4 (7:83919309) is located in the *EDIL3* gene (ENSBTAT00000121872.1, exon 11/11), and a (T) was deleted, causing a frameshift. The four lead SNPs identified matched the unique identifier number from the National Center for Biotechnology Information (NCBI 2025) SNP data base: rs518509552 for lead SNP 1, rs438544900 for lead SNP 2, rs450555472 for lead SNP 3, and rs470818992 for lead SNP 4. Lead SNPs were visually confirmed using IGV (Figures S1–S4). Exons and protein sequences of variants found in protein‐coding regions were verified using Ensembl (Dyer et al. 2025) and NCBI (Figures S5–S7).

Haplotypes were built with 20 upstream and 20 downstream variants in LD with each lead SNP with *r* ^2^ > 0.8 (Tables S2–S5). The associations between the haplotypes and GEBV for TO, VO, and EMBR were investigated. The variant (7:83173097) in high LD with lead SNP 1 showed four alternate alleles, most of which are repetitions of adenines and thymines. For this variant, sires with positive GEBV for VO, TO, and EMBR showed at least one copy of alternative allele 2 (A), while the other copy was either the reference allele (AAT), alternative allele 1 (AATAT), or the alternative allele 2 (A). For Lead SNP 2, four sires were heterozygous and eight were homozygous for the reference allele. However, no differences were found in the lead SNP 2 haplotypes when comparing bulls with high and low GEBV. For lead SNP 4, all three genotypes were identified among the 12 sires: homozygous for reference allele, heterozygous, and homozygous for the mutant allele. For lead SNP 3, all sires showed at least one copy of the mutant allele, and most were homozygous for this SNP. Plots showing recombination events were built with approximately 226 SNP markers (Figures S8–S19).

We explored the relationship between GEBV, phenotype, and sire genotype to verify how paternal genetics contributes to the productivity of oocytes and embryos in daughters and to get an initial insight of how the breed improvement program could be optimized with this new information. Average GEBV from the daughters was negative for all traits and genotype groups, except for EMBR for the heterozygous genotype of Lead SNP 4 (Table 3). For Lead SNP 1 (rs518509552), average GEBV of all traits for the daughters of heterozygous sires (C/G) was significantly lower (*p* < 0.05) than the average GEBV for the daughters of homozygous sires for the reference allele (C/C). Average number of embryos was also significantly lower (*p* = 2.306e‐05) for the daughters of heterozygous sires (C/G). For Lead SNP 2 (rs438544900), average GEBV and phenotype for all traits were significantly lower (*p* < 0.05) for the daughters of heterozygous sires (C/T) than for the daughters of homozygous sires for the reference allele (C/C). For Lead SNP 3 (rs450555472), average GEBV and phenotypes for all traits for the daughters of homozygous sires for the mutant allele (C/C) were significantly higher (*p* < 0.05) than the averages for the daughters of heterozygous sires (CTTTTT/C). For Lead SNP 4, average GEBV was significantly lower (*p* < 0.05) for the daughters of heterozygous sires (AT/A_) than for the daughters of both homozygous sires (reference and mutant), which did not differ from each other. It is important to note that although there was a large number of daughters and phenotypes in the homozygous group for the reference allele, there was only one bull with that genotype. However, for Lead SNP 4, the average number of viable oocytes was higher in both homozygous groups than for the heterozygous group, while the average number of total oocytes was higher for the reference homozygous, followed by the mutant homozygous and finally, the heterozygous.

**TABLE 3:** Analysis of variance for Genomic Estimated Breeding Value (GEBV) and phenotype of daughters according to sires’ genotype for each lead SNP in dairy Gir cattle.

Genotype	Lead SNP 1 (rs518509552)					
	C/C (wild)		C/G		G/G (mutation)	
	10 sire families		2 sire families		—	
	**Number of daughters**	**Number of phenotypes**	**Number of daughters**	**Number of phenotypes**	**Number of daughters**	**Number of phenotypes**
	4975	45 938	2267	22 067	—	—

	GEBV	Phenotypes	GEBV	Phenotypes	GEBV	Phenotypes	*p* GEBV	*p* Phenotype
Viable oocytes	−0.04^a^	15.76	−0.08^b^	15.78	—	—	2.266e–06	0.8415
Total oocytes	−0.04^a^	20.29	−0.08^b^	20.31	—	—	1.652e–07	0.8671
Embryos	−0.04^a^	4.11^a^	−0.07^b^	3.95^b^	—	—	2.462e–08	2.306e‐05

Genotype	Lead SNP 2 (rs438544900)					
	C/C (wild)		C/T		T/T (mutation)	
**Sires**	8 sire families		4 sire families		—	
	**Number of daughters**	**Number of phenotypes**	**Number of daughters**	**Number of phenotypes**	**Number of daughters**	**Number of phenotypes**
	3803	35 024	3439	32 981	—	—

	GEBV	Phenotypes	GEBV	Phenotypes	GEBV	Phenotypes	*p* GEBV	*p* Phenotype
Viable oocytes	−0.03^a^	15.97^a^	−0.08^b^	15.55^b^	—	—	1.084e–12	5.393e–06
Total oocytes	−0.04^a^	20.50^a^	−0.08^b^	20.08^b^	—	—	4.425e–09	0.0001587
Embryos	−0.001^a^	4.20^a^	−0.10^b^	3.90^b^	—	—	2.2e–16	2.2e–16

Genotype	Lead SNP 3 (rs450555472)					
	CTTTTT/CTTTTT (wild)		CTTTTT/C		C/C (mutation)	
**Sires**	—		2 sire families		10 sire families	
	**Number of daughters**	**Number of phenotypes**	**Number of daughters**	**Number of phenotypes**	**Number of daughters**	**Number of phenotypes**
	—	—	1033	9186	6209	58 819

	GEBV	Phenotypes	GEBV	Phenotypes	GEBV	Phenotypes	*p* GEBV	*p* Phenotype
Viable oocytes	—	—	–0.12^b^	15.32^b^	−0.04^a^	15.84^a^	1.189e–14	0.0001157
Total oocytes	—	—	−0.10^b^	19.97^b^	−0.05^a^	20.34^a^	2.15e–08	0.02217
Embryos	—	—	−0.15^b^	3.94^b^	−0.03^a^	4.08^a^	2.2e–16	0.00399

Genotype	Lead SNP 4 (rs470818992)					
	AT/AT (wild)		AT/A_		A_/A_ (mutation)	
**Sires**	1 sire family		5 sire families		6 sire families	
	**Number of daughters**	**Number of phenotypes**	**Number of daughters**	**Number of phenotypes**	**Number of daughters**	**Number of phenotypes**
	2088	20 589	2076	20 669	3078	26 747

	GEBV	Phenotypes	GEBV	Phenotypes	GEBV	Phenotypes	*p* GEBV	*p* Phenotype
**Viable oocytes**	–0.06^b^	16.08^a^	–0.01^a^	15.25^b^	–0.07^b^	15.93^a^	6.903e–14	5.023e–13
**Total oocytes**	–0.07^b^	20.64^a^	–0.02^a^	19.74^c^	–0.08^b^	20.46^b^	7.356e–14	1.045e–10
**Embryos**	–0.07^b^	4.03	0.02^a^	4.04	–0.08^b^	4.09	2.2e–16	0.3461

*Note:* Slash (/): allele division for that genotype; letters indicate statistical difference (*p* < 0.05) in rows between GEBV averages or phenotype averages according to *t*‐test.

Abbreviations: A: Adenine; C, Cytosine; G, Guanine; T, Thymine.

**FIGURE 1:**
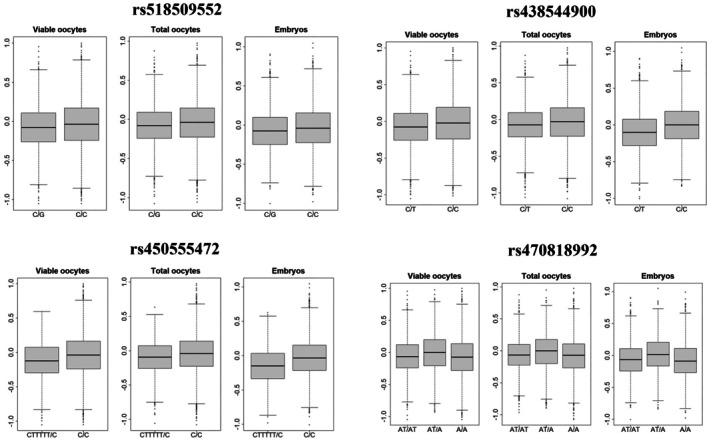
Boxplot of daughter GEBVs for the number of viable oocytes, total oocytes, and embryos, grouped by sire genotype for lead SNP 1 (rs518509552, substitution of a cytosine (C) by a guanine (G)), lead SNP 2 (rs438544900, substitution of a cytosine (C) by a thymine (T)), lead SNP 3 (rs450555472, deletion of five T), and lead SNP 4 (rs470818992, deletion of one T).

**FIGURE 2:**
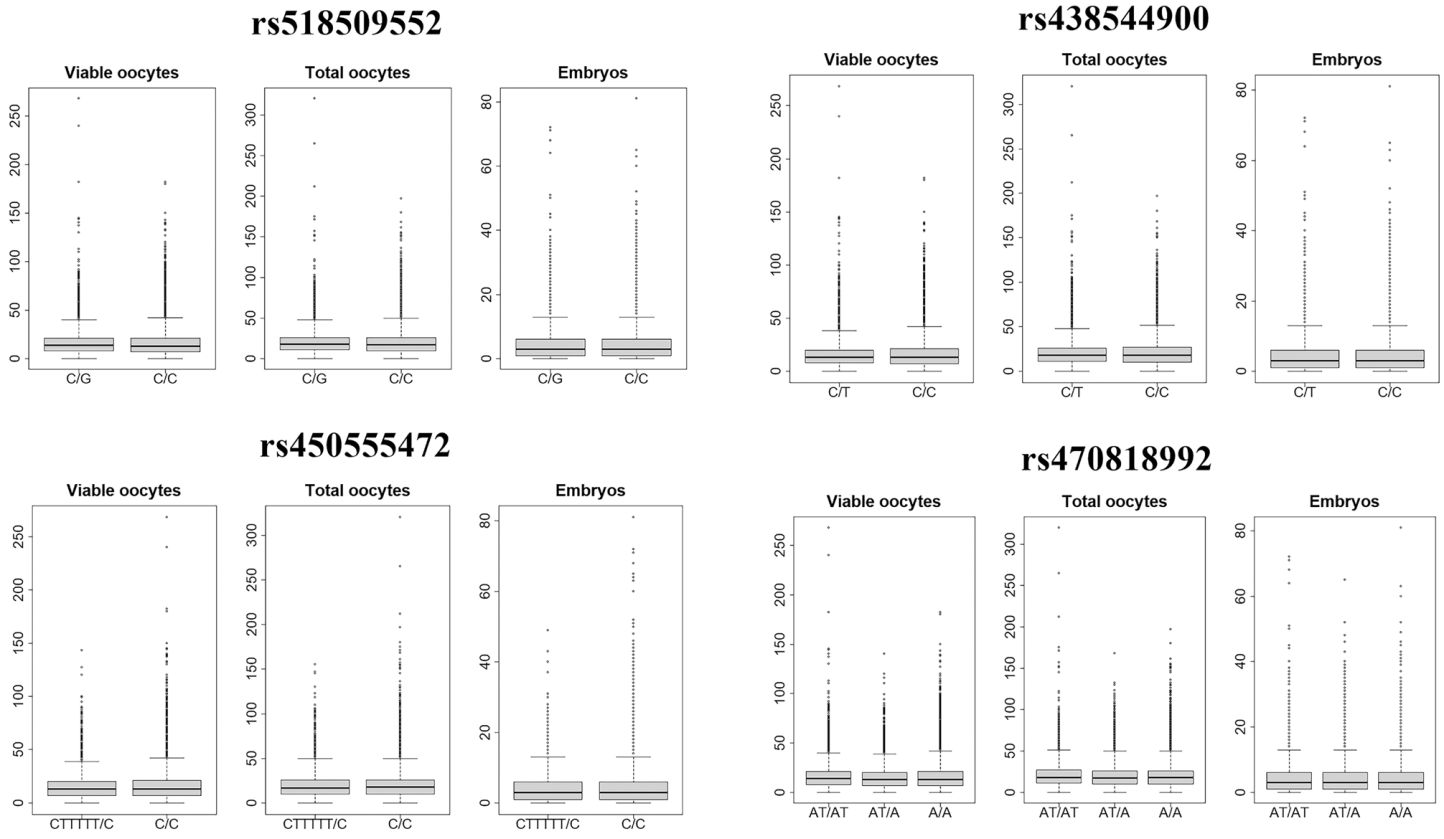
Boxplot of Gir daughter's phenotypes for the number of viable oocytes, total oocytes, and embryos, grouped by sire genotype for lead SNP 1 (rs518509552, substitution of a cytosine (C) by a guanine (G)), lead SNP 2 (rs438544900, substitution of a cytosine (C) by a thymine (T)), lead SNP 3 (rs450555472, deletion of five T), and lead SNP 4 (rs470818992, deletion of one T).

Most grouped daughters showed negative GEBV for the traits, similar to most of the sires' GEBV. The exceptions were only for number of embryos for the heterozygous (AT/A_) group of lead SNP 4. Boxplots for the average GEBVs of the daughters according to the sires' genotype for each lead SNP are available in Figure 1. Boxplots for the average phenotype of the daughters according to the sires' genotypes for each lead SNP are available in Figure 2.

## 4 Discussion

To address the lack of genomic variants associated with oocyte and embryo production, this study explored genomic variants of 12 Gir sires and we identified four mutations affecting the number of oocytes and embryos produced by their daughters. This study was conducted to identify mutations within a specific region on BTA7. Since these bulls show high PTA for milk production, they are widely used in dairy Gir and Girolando breeding programs and, therefore, they have a very high number of daughters in herds of both breeds in Brazil.

The four variants had statistical effects on phenotypes and GEBV for the number of oocytes and embryos in Gir, named lead SNPs. For Lead SNP 1 (rs518509552), the G mutant allele in the heterozygous was associated with a decrease in GEBV values, and although the average number of oocytes collected was not changed, the number of embryos obtained was decreased. This suggests an unfavorable association between the G allele and the studied traits. The same applies to the mutant allele T at lead SNP 2 (rs438544900), which was associated with a decrease in the average phenotype and GEBV for all traits in the heterozygous. On the other hand, in Lead SNP 3 (rs450555472), the mutant allele in homozygosity on the sires was associated with an increase in the average number of oocytes and embryos as well as with their average GEBV in the daughters. These results suggest that this regulatory region might provide a favorable effect on the traits when the mutant allele is inherited from both sire and dam. In Lead SNP 4 (rs470818992), it seems that the reference or the mutant allele alone are not associated with an increase or decrease in the average GEBV, but the interaction between both alleles in the heterozygous genotype is associated with a decrease in the average GEBV for all traits. This suggests a haploinsufficiency effect, which requires that both copies of the gene are normally functioning; otherwise, it could cause a reduction in the dosage of the encoded proteins (Johnson et al. 2019). For the phenotypes, it was also observed that heterozygosity was associated with a decrease in the average number of viable oocytes and total oocytes, reinforcing the possibility of interaction between both alleles. The statistical differences for the number of total oocytes might suggest interaction between alleles in heterozygosity for lead SNP 4, without dominance. The identified variants were searched in the literature, but to the best of our knowledge, they have not previously been associated with any livestock traits. Therefore, our discussion is based on mutations in the genes identified in our research affecting reproductive traits, as well as other traits, indicating a potential pleiotropic effect of some mutations. In this study, we propose these mutations affect the production of oocytes and embryos in Gir dairy cattle, but we recommend further functional studies of these mutations in future research.

Reproductive traits, such as the number of oocytes and embryos, are polygenic traits and therefore affected by several genomic regions (Rocha et al. 2024a). On BTA 7, for example, associations have been observed for the number of oocytes and embryos in dairy Gir cattle (Rocha et al. 2024a), calving interval and gestation length in Hanwoo cows (Haque et al. 2024), and calving ease and survival in Holstein cattle attributed to sire effects (Chen et al. 2022). These references suggest that BTA7 carries genes and mutations affecting several reproductive traits. Thus, the genetic correlation between oocyte/embryo production and other reproductive traits should be confirmed in future studies.

In our research, we found mutations in the sequence of *XRCC4*, *HAPLN1*, and *EDIL3* genes, which were identified in our previous study as associated with the number of oocytes and embryos (Rocha et al. 2024a). Comparing whole‐genome sequence of Nellore, Gir and Hereford breeds, Thambiraja et al. (2024) uncovered 1147 substitutions, 17 insertions, and 4 deletions exclusively in the *Bos indicus EDIL3* gene, which was linked to innate immunity. These findings show the large number of mutations that can be found in a single gene, highlighting that, in addition to being affected by several genes, polygenic traits can also be influenced by several mutations within a single gene. It is also important to highlight the possibility of pleiotropic effects, where mutations within the same gene trigger different physiological events, impacting more than one trait. For example, Singh et al. (2022) found significant SNPs in the sequence of *VCAN*, *HAPLN1* and *EDIL3* genes related to calcium content in the milk of Vrindavani cattle, a taurine‐indicine crossbred. This may suggest a genetic relationship between oocyte/embryo production and milk mineral content, which could be verified in future studies. Furthermore, *VCAN* was one of the genes uncovered in the predetermined region on BTA7 along with *XRCC4*, *HAPLN1*, and *EDIL3* (Rocha et al. 2024a). Although we did not find any mutations with high impact in the *VCAN* gene on EsembleVEP, some mutations in this gene were in linkage disequilibrium with the lead SNPs found in our study (Tables S2–S5). Also, the literature on these specific variants is scarce, with most studies focusing on larger Copy Number Variations (Boussaha et al. 2015; Mesbah‐Uddin et al. 2018).

Interestingly, *XRCC4* phosphorylation suppresses DNA double‐strand breaks repair to prevent genome instability during mitosis (Terasawa et al. 2014). A succession of mitotic processes occurs during the development from a fertilized oocyte to a fully formed embryo, which may explain the association of the *XRCC4* gene with the studied traits, possibly by preventing genome instability. This suggests that the mutation in *XRCC4* causing protein truncation may trigger instability during mitosis and lead to DNA double‐strand breaks, potentially impacting embryo development and the total number of embryos obtained. This is supported by strong expression of the *XRCC4* gene in granulosa cells and oocytes in rats (Talibova et al. 2024) and late embryonic lethality caused by *XRCC4* deficiency (Gao et al. 1998). Similarly, a recent study identified a stop‐codon variant in the *HAPLN1* gene associated with genuine empty follicle syndrome (Lledó et al. 2024).

The identification of stop codons, as seen with lead SNPs 1 and 2, may adversely affect economically important traits, exemplified by early pregnancy loss in cattle linked to homozygosity for specific haplotypes (Häfliger et al. 2021, 2022). It is also worth mentioning a nonsense mutation in the *APAF1* gene in Holstein sires that, when homozygous, results in a rare and fatal congenital anomaly that caused more than 500 000 spontaneous abortions worldwide over 30 years (Adams et al. 2016). For lead SNP 4, a deletion occurred, causing a frameshift and changing the reading frame of the genetic code, altering the entire amino acid sequence from that position. In addition to variants within genes, variants in intergenic regions, like lead SNP 3, can also affect the expression of phenotypes, as has been demonstrated for traits such as muscle development (Doyle et al. 2020) and milk production in cattle (Sanchez et al. 2017). The deletion of multiple thymines in lead SNP 3, located within a lncRNA, should be further explored to verify whether it functions as a regulatory region and to assess its impact on phenotypes.

Chen et al. (2022) found a significant SNP (position 83320343, ARS‐UCD1.2 assembly) on BTA7 (situated between lead SNP 1 and lead SNP 2 from our study), which was associated with sire calving ease and calving survival for Holstein cows and heifers, indicating *XRCC4* and *VCAN* as candidate genes for these traits. Since haplotypes in this region are largely conserved in our study, it is possible that the number of oocytes and embryos shows a genetic relationship with calving ease and survival. However, studies exploring this hypothesis are still limited. In an initial attempt to associate the effect of the lead SNPs with the studied traits, the average genomic values (GEBVs) of the daughters for each variant genetic group were analyzed. The results indicated that all the variants showed a significant impact on the production of oocytes and embryos at both the phenotypic and genomic levels. This opens opportunities for validation studies to confirm the presence of such variants in Gir and Girolando breeds and their actual effects.

Regarding haplotype results, sire 1 was the only sire homozygous for the lead SNP 4 reference allele. This sire also showed different haplotypes for lead SNP 1 and lead SNP 4 when compared to all the other sires. Sire 1 showed a negative GEBV for oocytes and embryos and is the sire with the greatest number of daughters. Most of the sires showed negative GEBV for TO, VO, and EMBR but, as Sire 1 is currently one of the most used Gir sires in Brazil, he has a greater chance to propagate his variants through the herds, which might impact negatively the production of oocytes and embryos. Since literature examples of variants causing great economic impact due to homozygous alleles resulting in pregnancy failure and estrus repetition (Adams et al. 2016), it is important to pay attention to such details, especially when studying the genotypes of the daughters.

The graphs of the region BTA7:82974837–83997563 were consistent with the LD results, as most variants in linkage disequilibrium with the lead SNPs showed values near to one, indicating how highly conserved this interval is around the variants with high impact and associated with number of oocytes and embryos. This finding suggests that the haplotype combination identified in the bulls is likely to be highly passed on to their daughters. Therefore, future studies should disentangle the architecture of this region to better understand the effects of the entire haplotype, not only on oocyte and embryo production but also on other traits. Moreover, analyzing this BTA7 interval in other indicine and taurine breeds may clarify its effects in other cattle groups and even shed light on the evolution of this genomic region in bovines.

Regarding the limitations of the study, although the *p*‐values were significantly high in our study, this significance may be disproportionately influenced by a single bull with a large number of daughters. The small number of parents per genotypic group can reduce statistical power. The ANOVA results should be interpreted as suggestive and require functional validation. In addition, there is a lack of studies exploring genomic regions associated with oocytes and embryos, which increases the need to explore such traits in other breeds. Also, we recommend sequencing of Gir cows and heifers and gene expression analysis to be carried out in future studies to evaluate the presence of those variants as well as their functional impact. We also recommend that structural variants such as copy number variations be explored in future studies to verify how they can affect gene function and its relation with oocyte and embryo production.

## 5 Conclusions

The variants rs518509552, rs438544900, rs450555472, and rs470818992 had a significant association with oocyte and embryo production in the Gir breed. The reference allele of rs518509552 and rs438544900 was associated with higher GEBV for oocyte and embryo production. In contrast, the mutant allele in a homozygous state for the rs450555472 variant was associated with higher average GEBV for the number of oocytes and embryos. Interestingly, the alleles of the rs470818992 variant appear to have an interaction effect in the heterozygous genotype, without dominance, but this requires further research since only one sire was homozygous for the reference allele in our study. Future studies should also evaluate the correlation between the production of oocytes and embryos and milk PTA, not only in Gir but also in other indicine and taurine breeds. Finally, the expression of the genes with different genotypes for the variants identified should be verified and compared to the effects on Gir donors and their respective oocytes and embryos.

## Funding

This work was supported by Conselho Nacional de Desenvolvimento Científico e Tecnológico, 150320/2024‐8. Empresa Brasileira de Pesquisa Agropecuária, SEG 02.13.05.011.00.00. Fundação de Amparo à Pesquisa do Estado de Minas Gerais, APQ‐02750‐23. Coordenação de Aperfeiçoamento de Pessoal de Nível Superior, PROEX 88887.844747/2023‐00, 88887.189271/2025‐00.

## Ethics Statement

The authors have nothing to report.

## Conflicts of Interest

The authors declare no conflicts of interest.

## Supporting information


**Figure S1:** Integrative Genomics Viewer (IGV) image from a 51 Kb region around rs518509552 (A) and zoom in rs518509552 (B).
**Figure S2:** Integrative Genomics Viewer (IGV) image from a 51 Kb region around rs438544900 (A) and zoom in rs438544900 (B).
**Figure S3:** Integrative Genomics Viewer (IGV) image from a 51 Kb region around rs450555472 (A) and zoom in rs450555472 (B).
**Figure S4:** Integrative Genomics Viewer (IGV) image from a 51 Kb region around rs470818992 (A) and zoom in rs470818992 (B).
**Figure S5:** Identification of the XRCC4 p.S343* mutation rs518509552. (a) Genomic region adapted from Genome Data Viewer ( *Bos taurus* Genome assembly ARS‐UCD1.2). Genes are shown with arrows indicating their reading direction and position. The box indicates the gene XRCC4. The dashed lines zoom into the region to the next image. (b) This image was adapted from Ensembl and shows schematic structure for *XRCC4* transcript ENSBTAT00000131234, including all 8 exons marked by vertical bars. The mutation was identified in exon 8. (c) The aminoacid sequence of *XRCC4* exon 8 shows the position with the stop‐gain mutation, indicated by X in red. Amino acids within the parentheses were truncated from exon 8, presumed deleted in individuals with the p.S343* mutation. The box with an up arrow below the sequence indicates the triplet that should be read, and the letter in red is the substitution in the Forward sequence.
**Figure S6:** Identification of the HAPLN1 p.R234* mutation rs438544900. (a) Genomic region adapted from Genome Data Viewer ( *Bos taurus* Genome assembly ARS‐UCD1.2). Genes are shown with arrows indicating their reading direction and position. The box indicates the gene HAPLN1. The dashed lines zoom into the region to the next image. (b) This image was adapted from Ensembl and shows schematic structure for *HAPLN1* transcript ENSBTAT00000098839, including all 2 exons marked by vertical bars. The mutation was identified in exon 2. (c) The aminoacid sequence of *HAPLN1* exon 2 shows the position with the stop‐gain mutation, indicated by X in red. Amino acids within the parentheses were truncated from exon 2, presumed deleted in individuals with the p.R234* mutation. The box with an up arrow below the sequence indicates the triplet that should be read, and the letter in red is the substitution in the Forward sequence.
**Figure S7:** Identification of the *EDIL3 c.1163del* mutation rs470818992. (a) Genomic region adapted from Genome Data Viewer ( *Bos taurus* Genome assembly ARS‐UCD1.2). Genes are shown with arrows indicating their reading direction and position. The box indicates the gene *EDIL3*. The dashed lines zoom into the region to the next image. (b) This image was adapted from Ensembl and shows schematic structure for *EDIL3* transcript ENSBTAT00000121872, including all 11 exons marked by vertical bars. The mutation was identified in exon 11. (c) The aminoacid sequence of *EDIL3* exon 11 shows the position with the deletion, indicated by X in red. Amino acids within the parentheses had its reading frame altered from the deletion point on exon 11, presumably forming a different protein in individuals with the *c.1163del* mutation. The box with an up arrow below the sequence indicates the triplet that should be read, and the letter in red is the deletion in the Forward sequence.
**Figure S8:** Hsphase Imageplot of recombination events on BTA 7 (A) and on 82 974 837‐83 997 563 interval ( *Bos taurus* Genome assembly ARS‐UCD1.2) (B) for sire's 1 daughters.
**Figure S9:** Hsphase Imageplot of recombination events on BTA 7 (A) and on 82 974 837‐83 997 563 interval ( *Bos taurus* Genome assembly ARS‐UCD1.2) (B) for sire's 2 daughters.
**Figure S10:** Hsphase Imageplot of recombination events on BTA 7 (A) and on 82 974 837‐83 997 563 interval ( *Bos taurus* Genome assembly ARS‐UCD1.2) (B) for sire's 3 daughters.
**Figure S11:** Hsphase Imageplot of recombination events on BTA 7 (A) and on 82 974 837‐83 997 563 interval ( *Bos taurus* Genome assembly ARS‐UCD1.2) (B) for sire's 4 daughters.
**Figure S12:** Hsphase Imageplot of recombination events on BTA 7 (A) and on 82 974 837‐83 997 563 interval ( *Bos taurus* Genome assembly ARS‐UCD1.2) (B) for sire's 5 daughters.
**Figure S13:** Hsphase Imageplot of recombination events on BTA 7 (A) and on 82 974 837‐83 997 563 interval ( *Bos taurus* Genome assembly ARS‐UCD1.2) (B) for sire's 6 daughters.
**Figure S14:** Hsphase Imageplot of recombination events on BTA 7 (A) and on 82 974 837‐83 997 563 interval ( *Bos taurus* Genome assembly ARS‐UCD1.2) (B) for sire's 7 daughters.
**Figure S15:** Hsphase Imageplot of recombination events on BTA 7 (A) and on 82 974 837‐83 997 563 interval ( *Bos taurus* Genome assembly ARS‐UCD1.2) (B) for sire's 8 daughters.
**Figure S16:** Hsphase Imageplot of recombination events on BTA 7 (A) and on 82 974 837‐83 997 563 interval ( *Bos taurus* Genome assembly ARS‐UCD1.2) (B) for sire's 9 daughters.
**Figure S17:** Hsphase Imageplot of recombination events on BTA 7 (A) and on 82 974 837‐83 997 563 interval ( *Bos taurus* Genome assembly ARS‐UCD1.2) (B) for sire's 10 daughters.
**Figure S18:** Hsphase Imageplot of recombination events on BTA 7 (A) and on 82 974 837‐83 997 563 interval ( *Bos taurus* Genome assembly ARS‐UCD1.2) (B) for sire's 11 daughters.
**Figure S19:** Hsphase Imageplot of recombination events on BTA 7 (A) and on 82 974 837‐83 997 563 interval ( *Bos taurus* Genome assembly ARS‐UCD1.2) (B) for sire's 12 daughters.

**Table S1:** Descriptive summary of consequences for insertions and deletions (Indels) and Single Nucleotide Polymorphism (SNP) identified in a Variant Call Format file inside region 7:82974837–83997 563 of 12 Gir sires.
**Table S2:** Double strand haplotypes^1^ built for 12 Gir sires with 20 upstream and 20 downstream variants with *r* ^2^ > 0.8 from the LeadSNP 1 (rs518509552, substitution of a cytosine by a guanine).
**Table S3:** Double strand haplotypes^1^ built for 12 Gir sires with 20 upstream and 20 downstream variants with *r* ^2^ > 0.8 from the LeadSNP 2 (rs438544900, substitution of a cytosine by a thymine).
**Table S4:** Double strand haplotypes^1^ built for 12 Gir sires with 20 upstream and 20 downstream variants with *r* ^2^ > 0.8 from the LeadSNP 3 (rs450555472, deletion of five thymines).
**Table S5:** Double strand haplotypes^1^ built for 12 Gir sires with 20 upstream and 20 downstream variants with *r* ^2^ > 0.8 from the LeadSNP 4 (rs470818992, deletion of one thymine).

## Data Availability

Data are commercially sensitive and are available under reasonable request from Embrapa Dairy Cattle Research Center. Data will only be available after signing a Material Transfer Agreement with restrictions on the use of the data, such as confidentiality.
