# Supplementary material for: Genomic Variants Associated With Oocyte and Embryo Production in Dairy Gir Cattle
Source: Anim Genet. 2026 Apr 12;57(2):e70098. doi: 10.1002/age.70098 (PMC13070668; doi:10.1002/age.70098)
Supplement: Supplementary file 1 — Figure S1: Integrative Genomics Viewer (IGV) image from a 51 Kb region around rs518509552 (A) and zoom in rs518509552 (B). Figure S2: Integrative Genomics Viewer (IGV) image from a 51 Kb region around rs438544900 (A) and zoom in rs438544900 (B). Figure S3: Integrative Genomics Viewer (IGV) image from a 51 Kb region around rs450555472 (A) and zoom in rs450555472 (B). Figure S4: Integrative Genomics Viewer (IGV) image from a 51 Kb region around rs470818992 (A) and zoom in rs470818992 (B). Figure S5: Identification of the XRCC4 p.S343* mutation rs518509552. (a) Genomic region adapted from Genome Data Viewer ( Bos taurus Genome assembly ARS‐UCD1.2). Genes are shown with arrows indicating their reading direction and position. The box indicates the gene XRCC4. The dashed lines zoom into the region to the next image. (b) This image was adapted from Ensembl and shows schematic structure for XRCC4 transcript ENSBTAT00000131234, including all 8 exons marked by vertical bars. The mutation was identified in exon 8. (c) The aminoacid sequence of XRCC4 exon 8 shows the position with the stop‐gain mutation, indicated by X in red. Amino acids within the parentheses were truncated from exon 8, presumed deleted in individuals with the p.S343* mutation. The box with an up arrow below the sequence indicates the triplet that should be read, and the letter in red is the substitution in the Forward sequence. Figure S6: Identification of the HAPLN1 p.R234* mutation rs438544900. (a) Genomic region adapted from Genome Data Viewer ( Bos taurus Genome assembly ARS‐UCD1.2). Genes are shown with arrows indicating their reading direction and position. The box indicates the gene HAPLN1. The dashed lines zoom into the region to the next image. (b) This image was adapted from Ensembl and shows schematic structure for HAPLN1 transcript ENSBTAT00000098839, including all 2 exons marked by vertical bars. The mutation was identified in exon 2. (c) The aminoacid sequence of HAPLN1 exon 2 shows the positio [file AGE-57-0-s001.docx]

**Supplementary File 1**


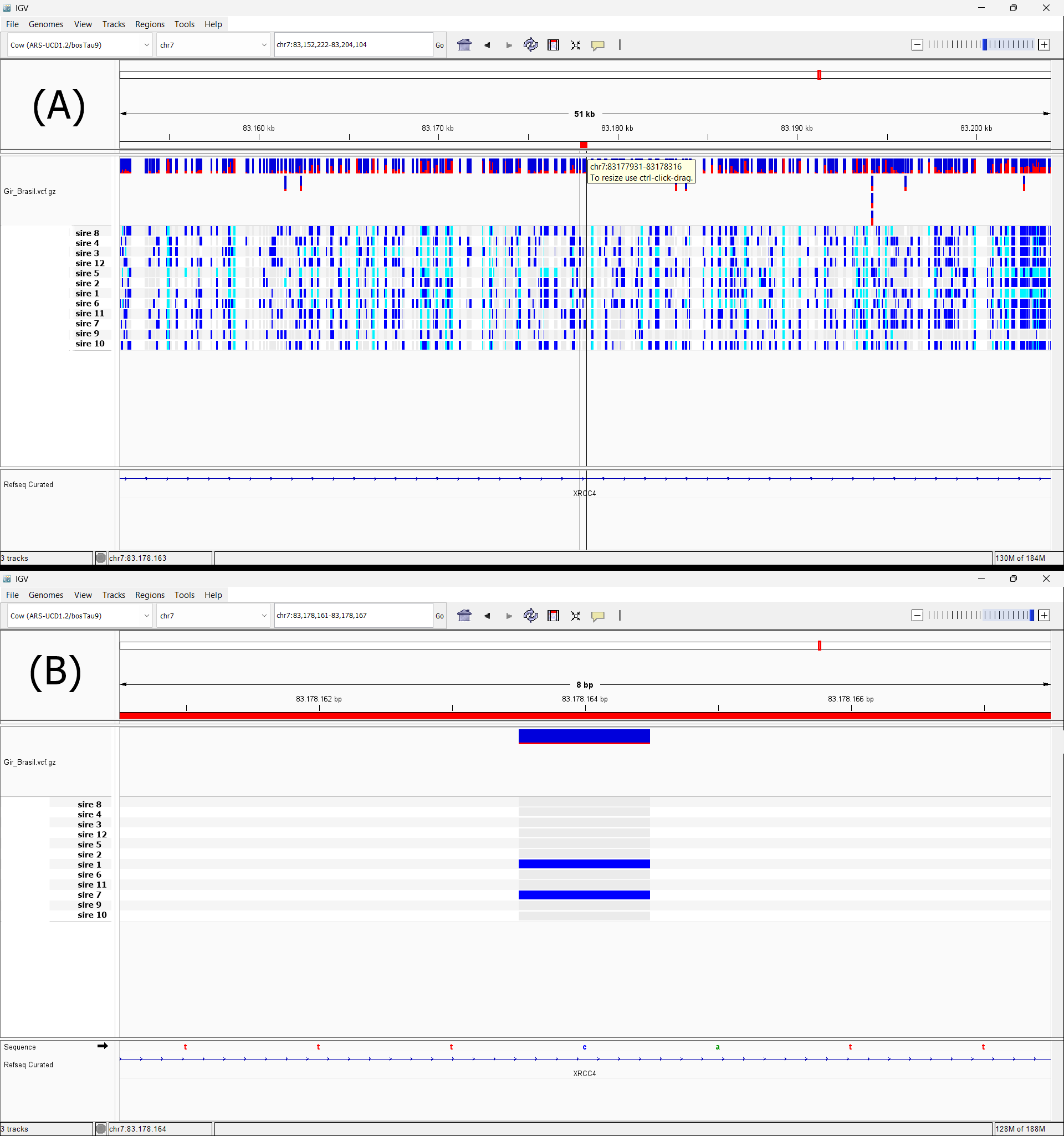
Supplementary Figure S1. Integrative Genomics Viewer (IGV) image from a 51 Kb region around rs518509552 (A) and zoom in rs518509552 (B).


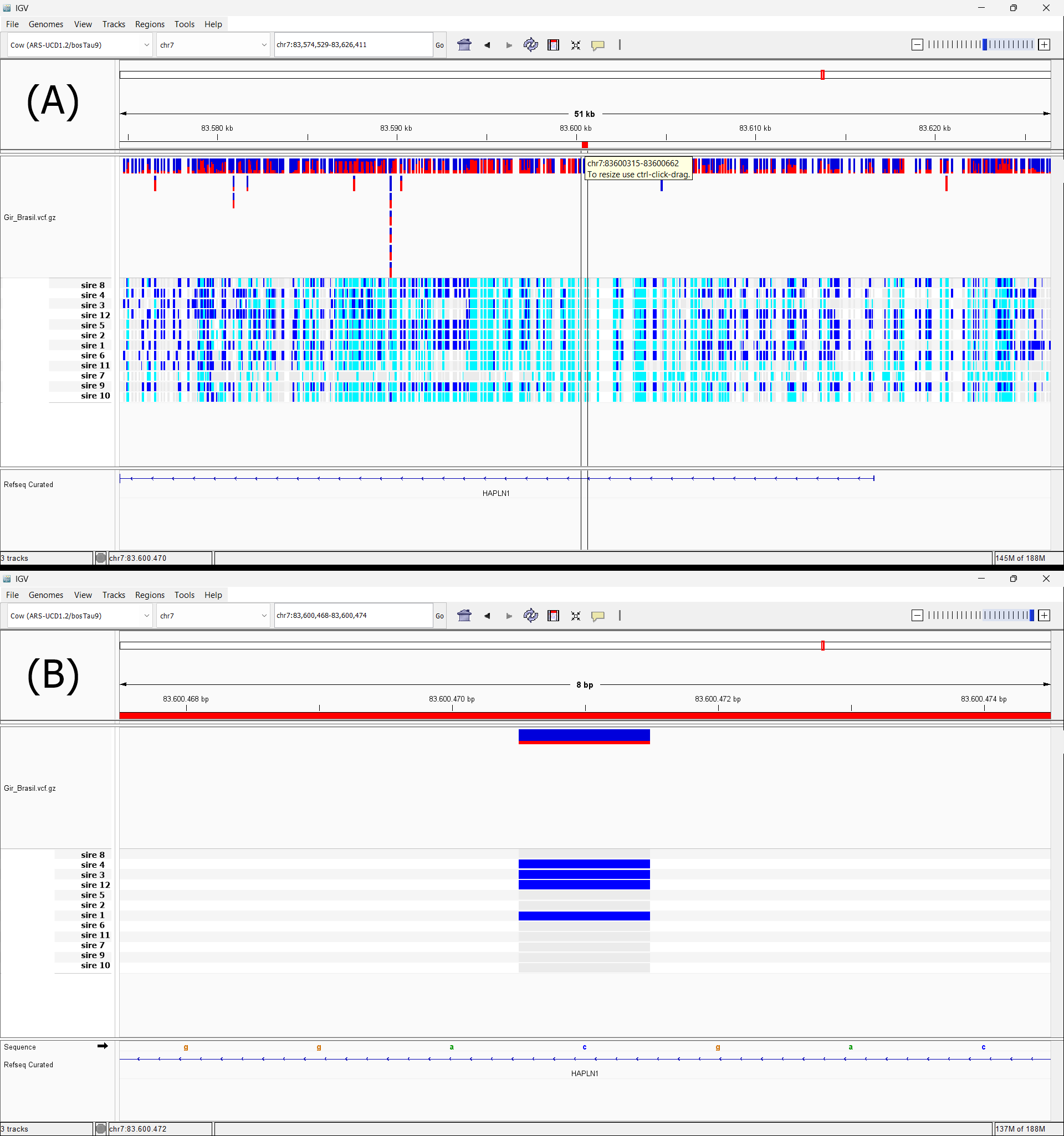
Supplementary Figure S2. Integrative Genomics Viewer (IGV) image from a 51 Kb region around rs438544900 (A) and zoom in rs438544900 (B).


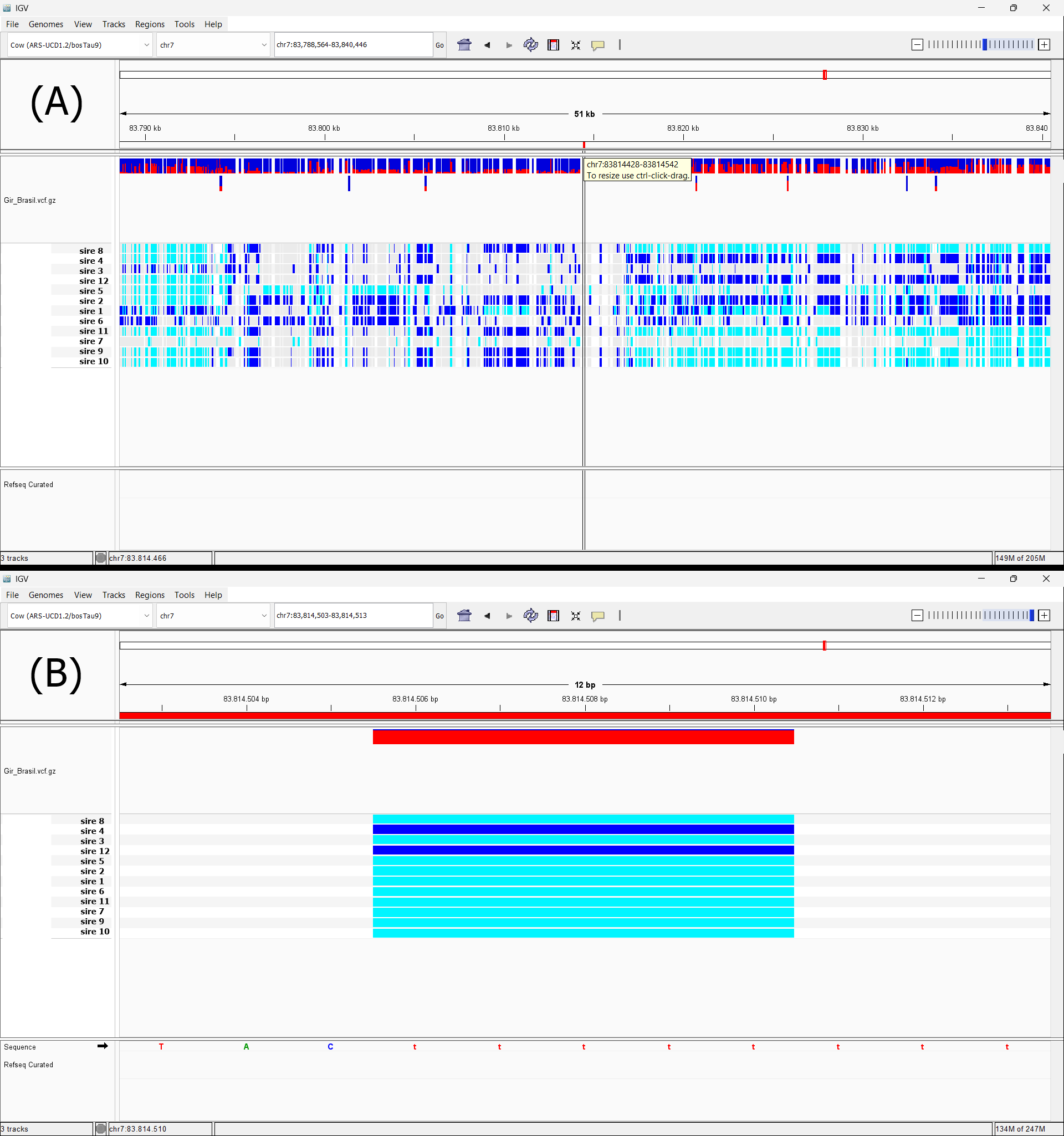
Supplementary Figure S3. Integrative Genomics Viewer (IGV) image from a 51 Kb region around rs450555472 (A) and zoom in rs450555472 (B).


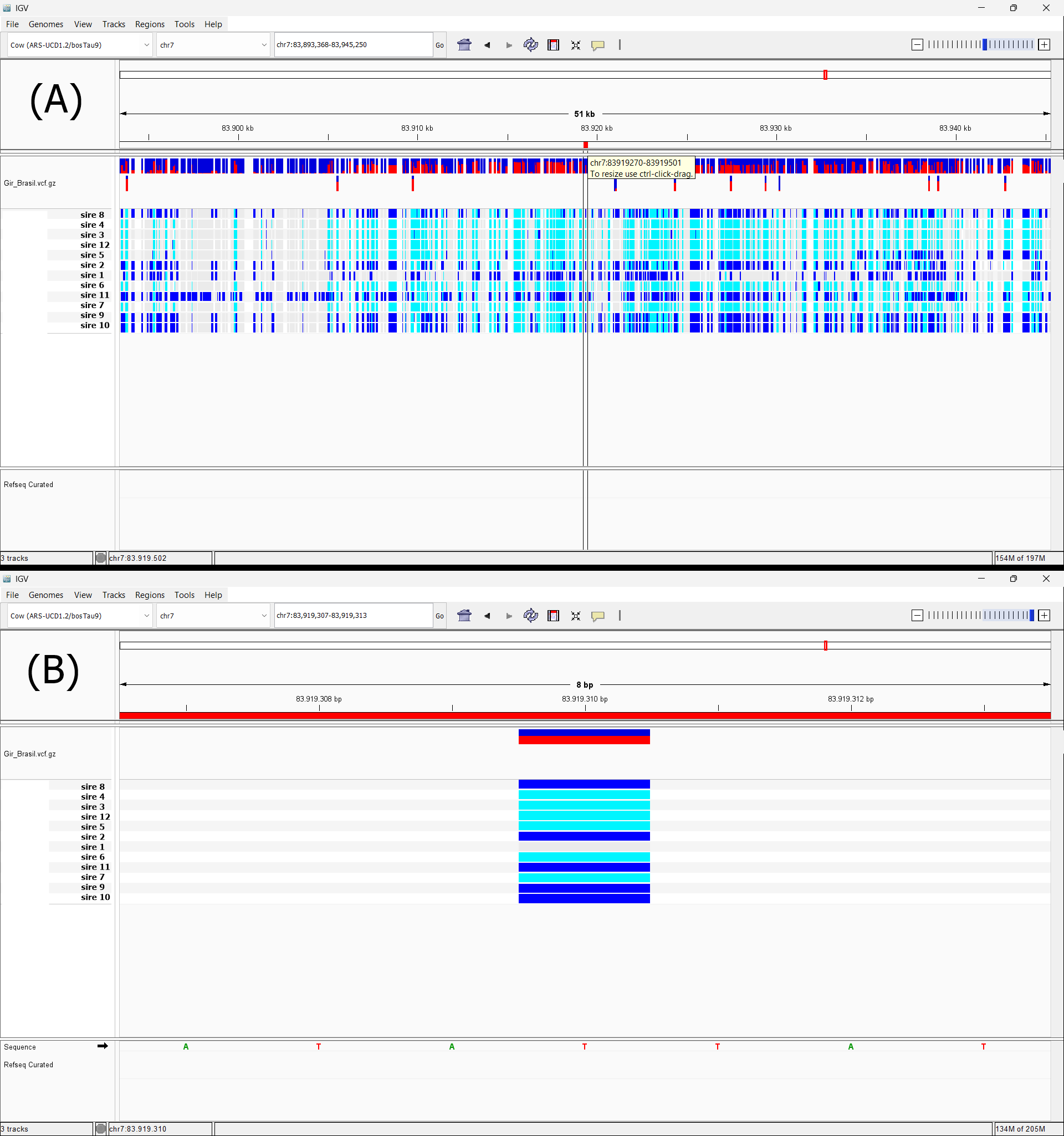
Supplementary Figure S4. Integrative Genomics Viewer (IGV) image from a 51 Kb region around rs470818992 (A) and zoom in rs470818992 (B).


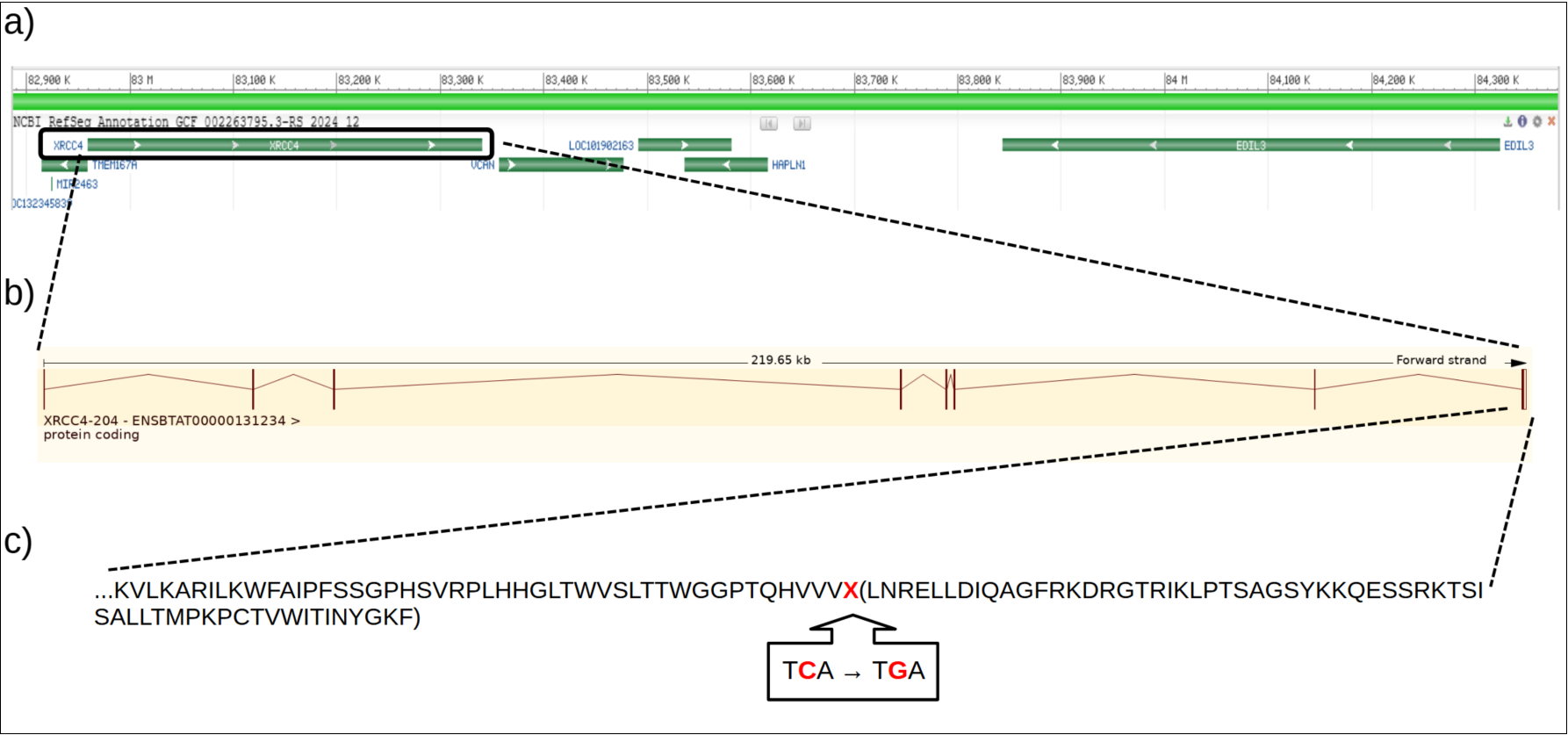
Supplementary Figure S5. Identification of the *XRCC4 p.S343** mutation rs518509552. (a) Genomic region adapted from Genome Data Viewer (*Bos taurus* Genome assembly ARS-UCD1.2). Genes are shown with arrows indicating their reading direction and position. The box indicates the gene XRCC4. The dashed lines zoom into the region to the next image. (b) This image was adapted from Ensembl and shows schematic structure for *XRCC4* transcript ENSBTAT00000131234*,* including all 8 exons marked by vertical bars. The mutation was identified in exon 8. (c) The aminoacid sequence of *XRCC4* exon 8 shows the position with the stop-gain mutation, indicated by X in red. Amino acids within the parentheses were truncated from exon 8, presumed deleted in individuals with the *p.S343** mutation. The box with an up arrow below the sequence indicates the triplet that should be read, and the letter in red is the substitution in the Forward sequence.


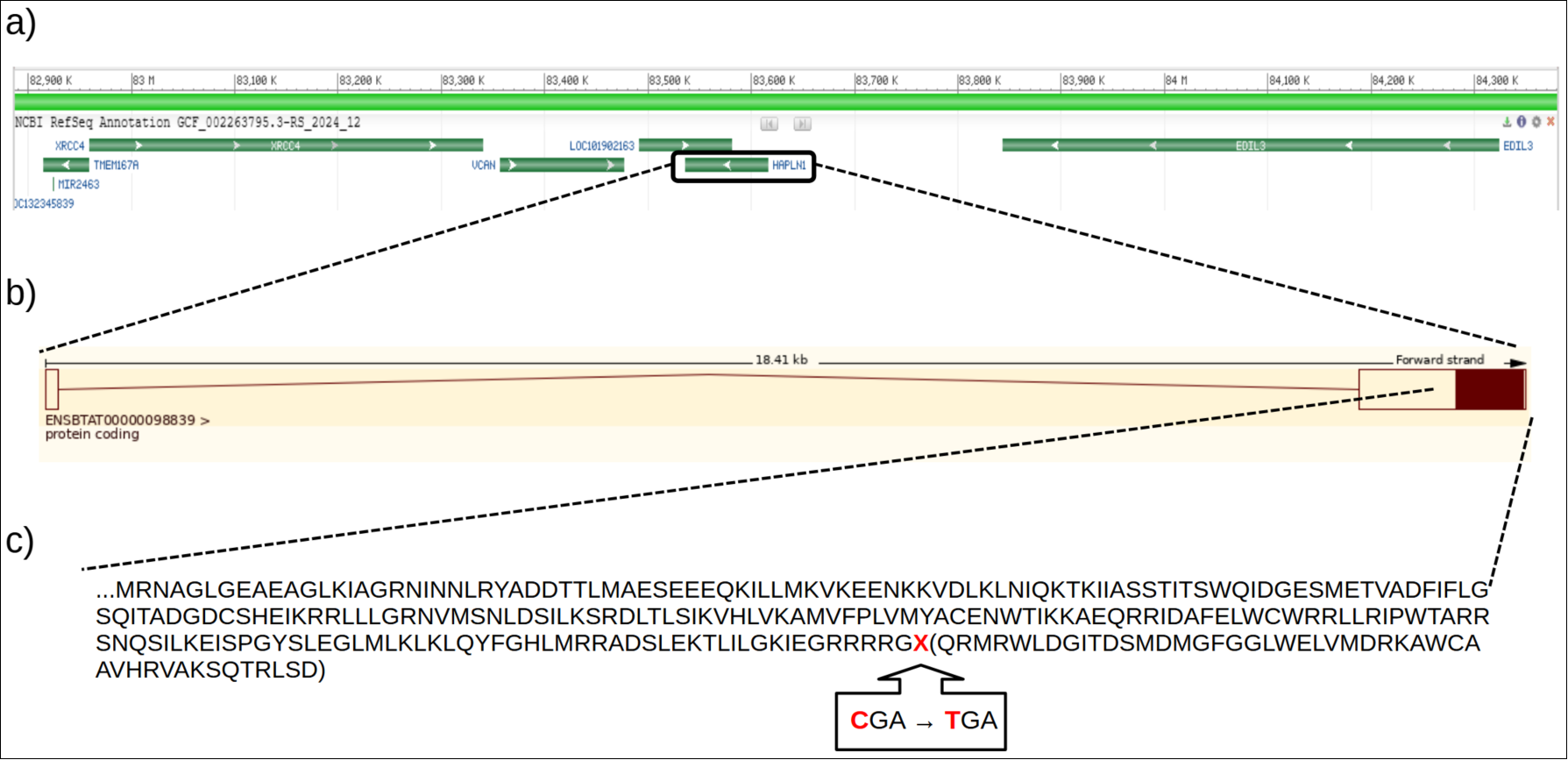
Supplementary Figure S6. Identification of the *HAPLN1 p.R234** mutation rs438544900. (a) Genomic region adapted from Genome Data Viewer (*Bos taurus* Genome assembly ARS-UCD1.2). Genes are shown with arrows indicating their reading direction and position. The box indicates the gene HAPLN1. The dashed lines zoom into the region to the next image. (b) This image was adapted from Ensembl and shows schematic structure for *HAPLN1* transcript ENSBTAT00000098839*,* including all 2 exons marked by vertical bars. The mutation was identified in exon 2. (c) The aminoacid sequence of *HAPLN1* exon 2 shows the position with the stop-gain mutation, indicated by X in red. Amino acids within the parentheses were truncated from exon 2, presumed deleted in individuals with the *p.R234** mutation. The box with an up arrow below the sequence indicates the triplet that should be read, and the letter in red is the substitution in the Forward sequence.


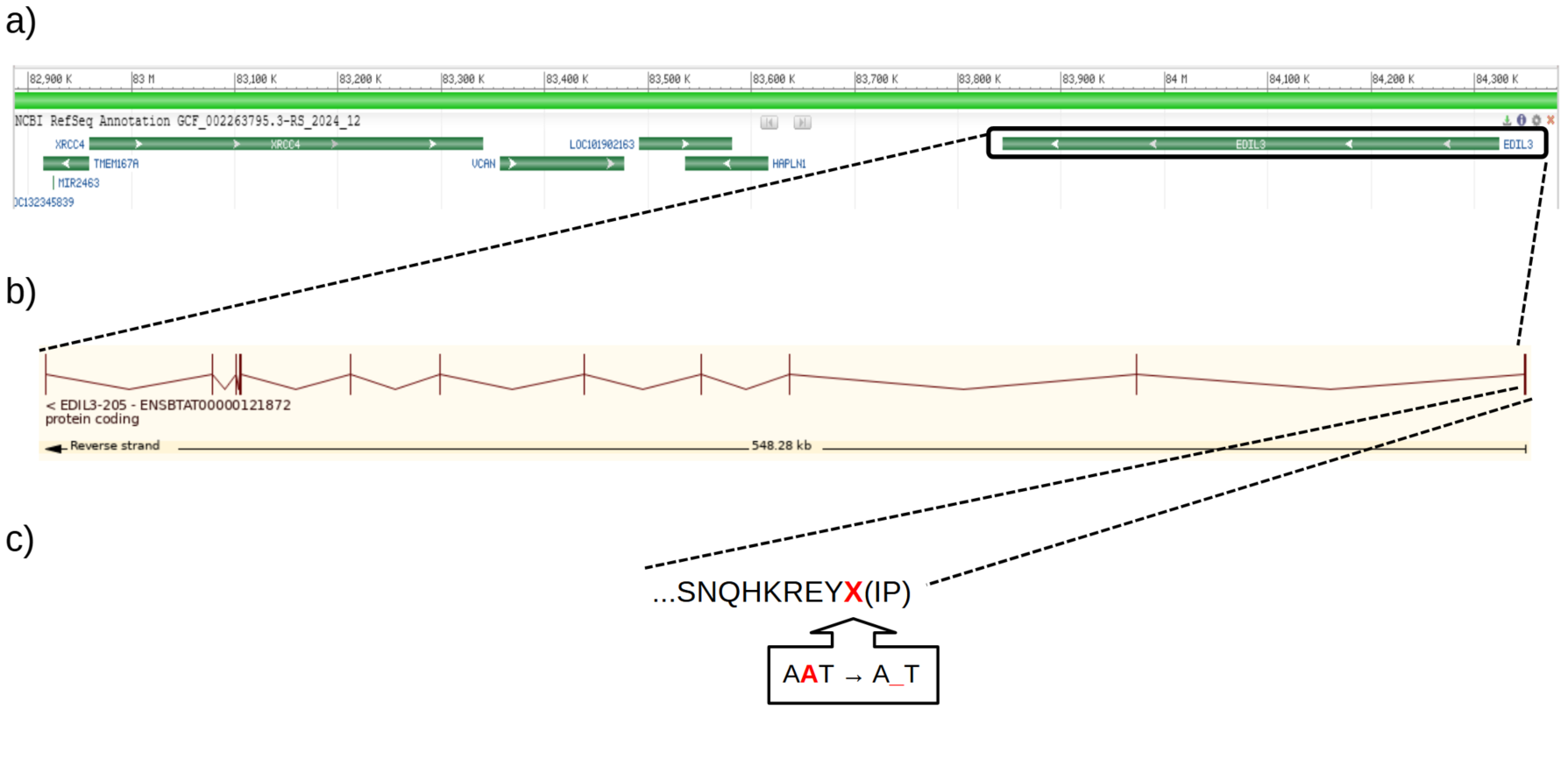
Supplementary Figure S7. Identification of the *EDIL3 c.1163del* mutation rs470818992. (a) Genomic region adapted from Genome Data Viewer (*Bos taurus* Genome assembly ARS-UCD1.2). Genes are shown with arrows indicating their reading direction and position. The box indicates the gene *EDIL3*. The dashed lines zoom into the region to the next image. (b) This image was adapted from Ensembl and shows schematic structure for *EDIL3* transcript ENSBTAT00000121872*,* including all 11 exons marked by vertical bars. The mutation was identified in exon 11. (c) The aminoacid sequence of *EDIL3* exon 11 shows the position with the deletion, indicated by X in red. Amino acids within the parentheses had its reading frame altered from the deletion point on exon 11, presumably forming a different protein in individuals with the *c.1163del* mutation. The box with an up arrow below the sequence indicates the triplet that should be read, and the letter in red is the deletion in the Forward sequence.


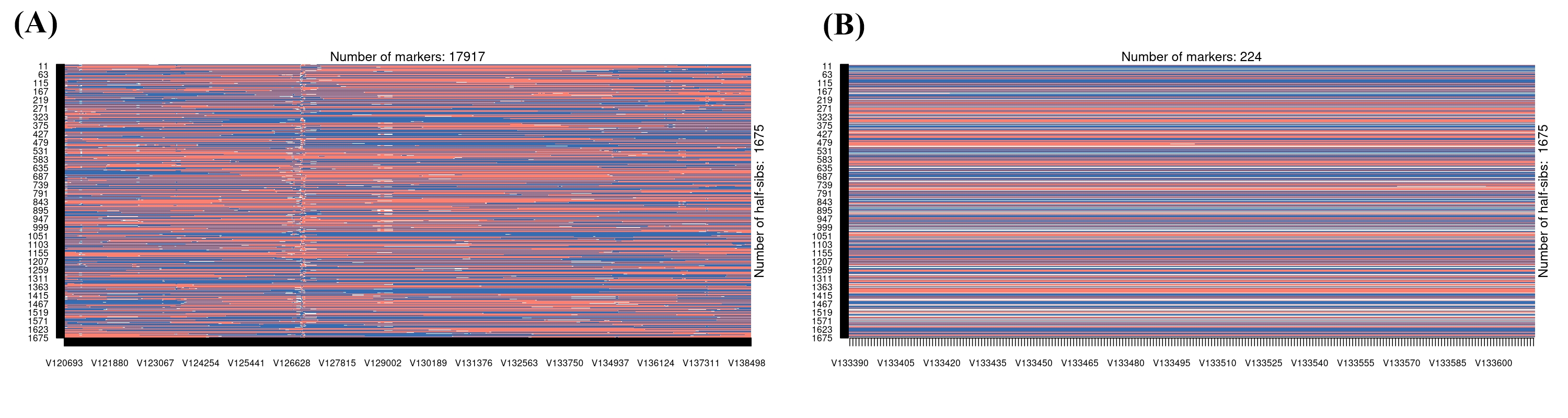
Supplementary Figure S8. Hsphase Imageplot of recombination events on BTA 7 (A) and on 82,974,837-83,997,563 interval (*Bos taurus* Genome assembly ARS-UCD1.2) (B) for sire’s 1 daughters.


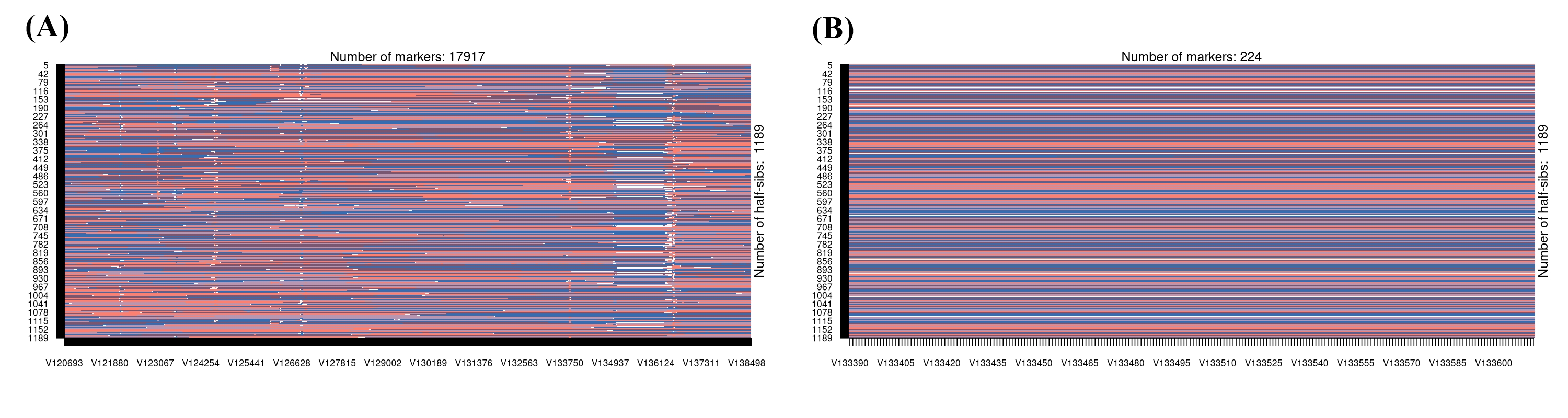
Supplementary Figure S9. Hsphase Imageplot of recombination events on BTA 7 (A) and on 82,974,837-83,997,563 interval (*Bos taurus* Genome assembly ARS-UCD1.2) (B) for sire’s 2 daughters.


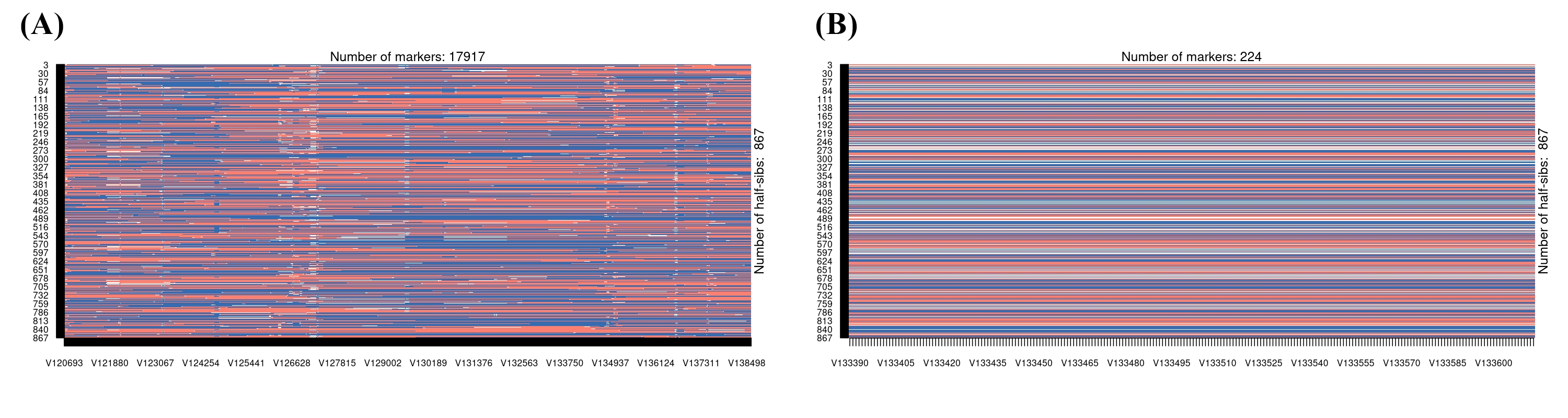
Supplementary Figure S10. Hsphase Imageplot of recombination events on BTA 7 (A) and on 82,974,837-83,997,563 interval (*Bos taurus* Genome assembly ARS-UCD1.2) (B) for sire’s 3 daughters.


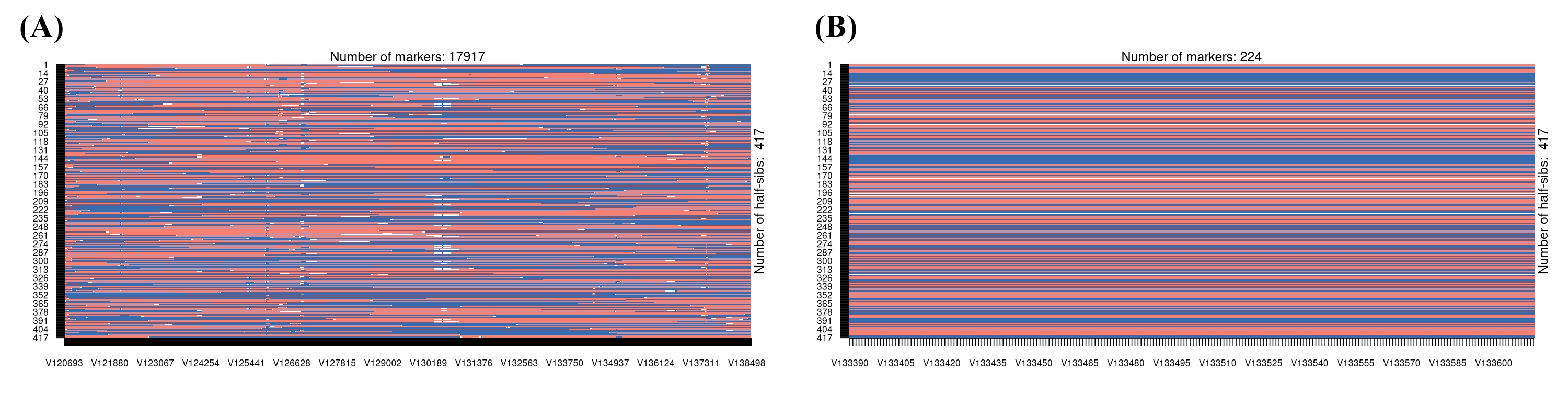
Supplementary Figure S11. Hsphase Imageplot of recombination events on BTA 7 (A) and on 82,974,837-83,997,563 interval (*Bos taurus* Genome assembly ARS-UCD1.2) (B) for sire’s 4 daughters.


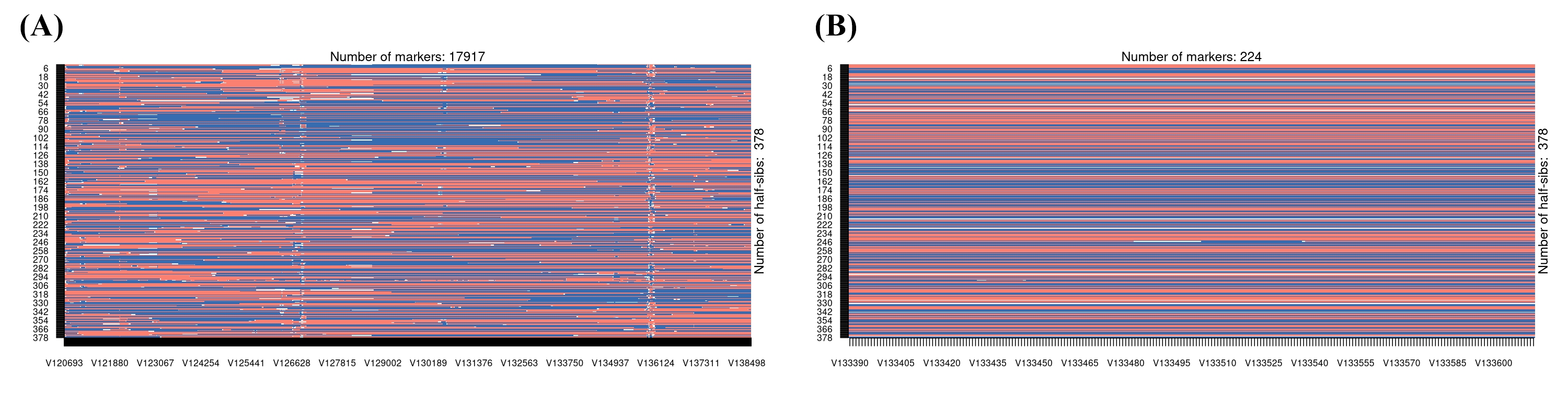
Supplementary Figure S12. Hsphase Imageplot of recombination events on BTA 7 (A) and on 82,974,837-83,997,563 interval (*Bos taurus* Genome assembly ARS-UCD1.2) (B) for sire’s 5 daughters.


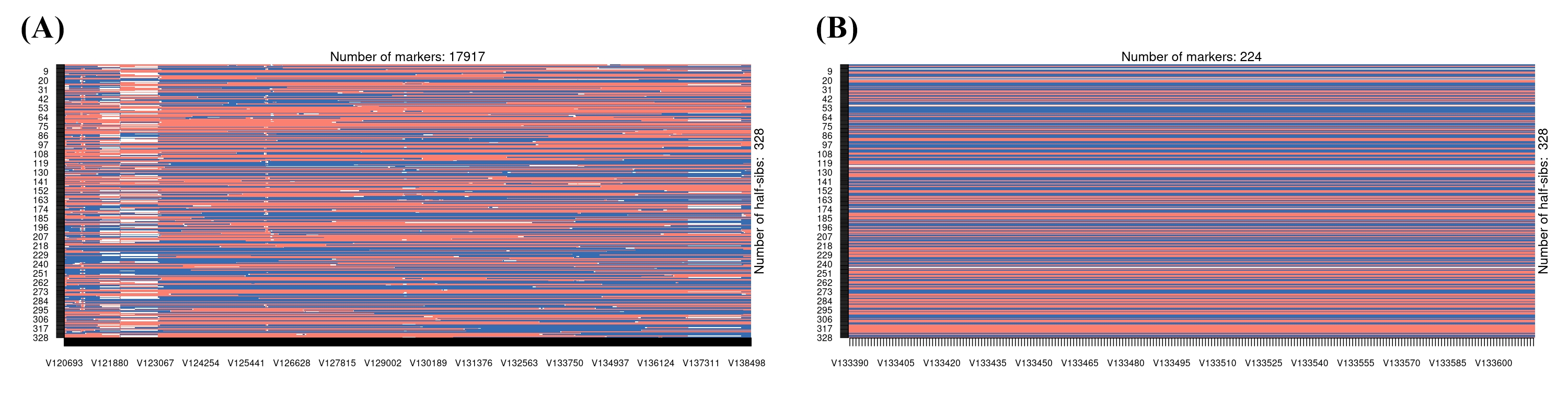
Supplementary Figure S13. Hsphase Imageplot of recombination events on BTA 7 (A) and on 82,974,837-83,997,563 interval (*Bos taurus* Genome assembly ARS-UCD1.2) (B) for sire’s 6 daughters.


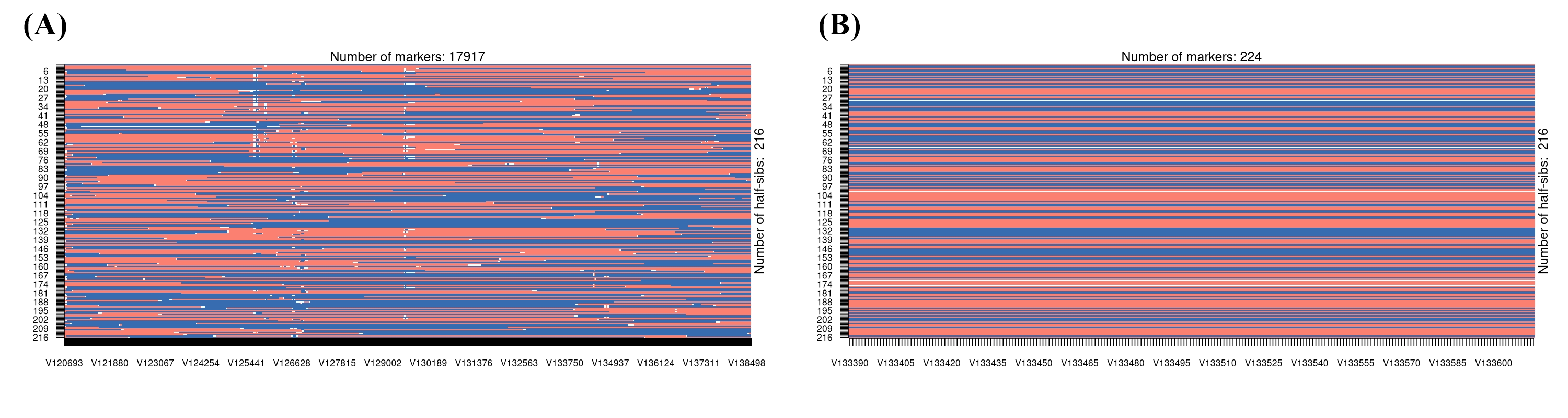
Supplementary Figure S14. Hsphase Imageplot of recombination events on BTA 7 (A) and on 82,974,837-83,997,563 interval (*Bos taurus* Genome assembly ARS-UCD1.2) (B) for sire’s 7 daughters.


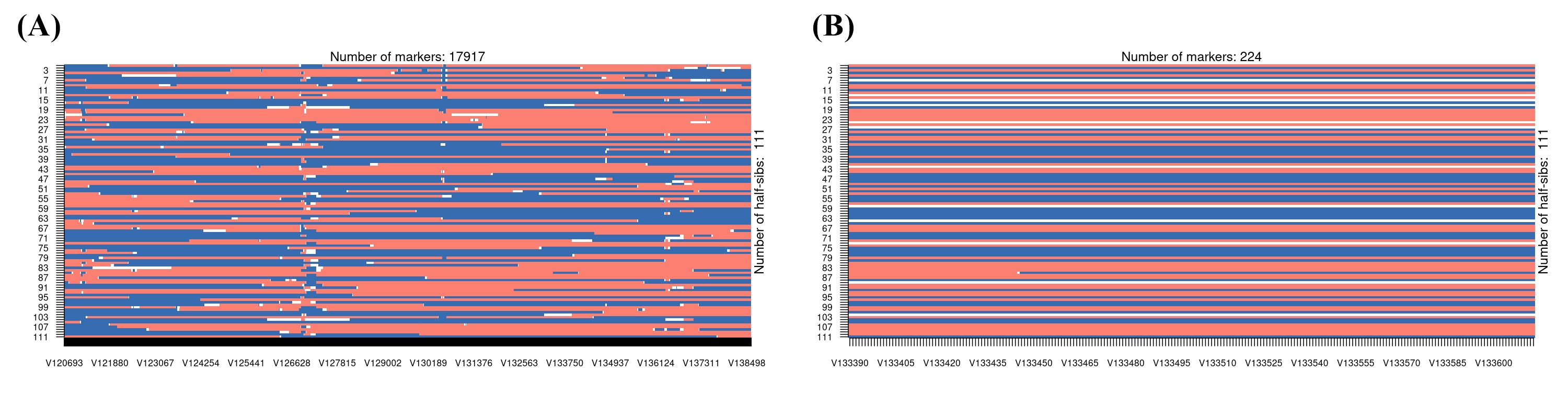
Supplementary Figure S15. Hsphase Imageplot of recombination events on BTA 7 (A) and on 82,974,837-83,997,563 interval (*Bos taurus* Genome assembly ARS-UCD1.2) (B) for sire’s 8 daughters.


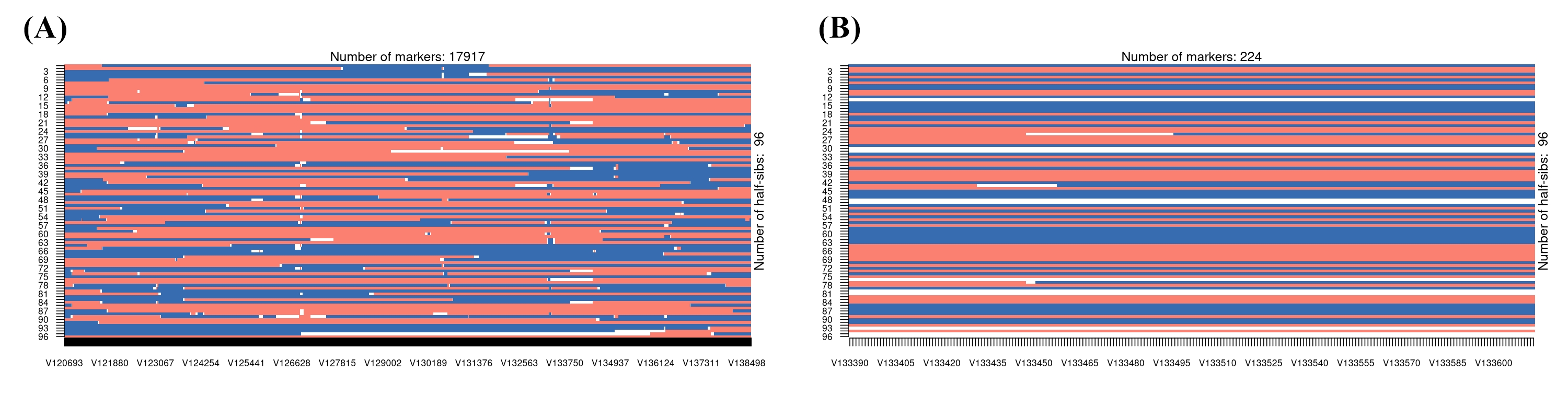
Supplementary Figure S16. Hsphase Imageplot of recombination events on BTA 7 (A) and on 82,974,837-83,997,563 interval (*Bos taurus* Genome assembly ARS-UCD1.2) (B) for sire’s 9 daughters.


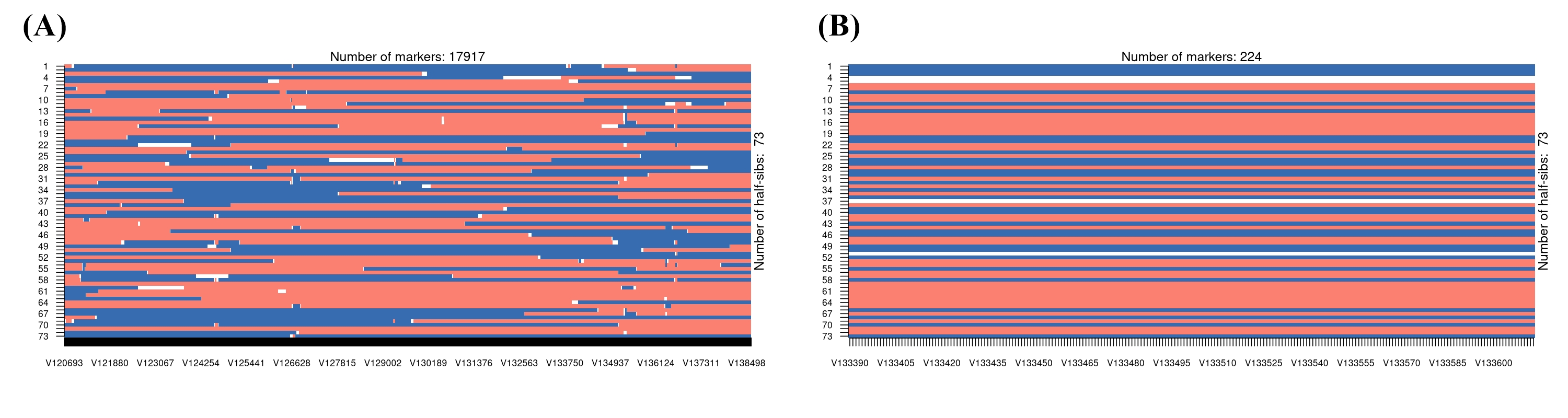
Supplementary Figure S17. Hsphase Imageplot of recombination events on BTA 7 (A) and on 82,974,837-83,997,563 interval (*Bos taurus* Genome assembly ARS-UCD1.2) (B) for sire’s 10 daughters.


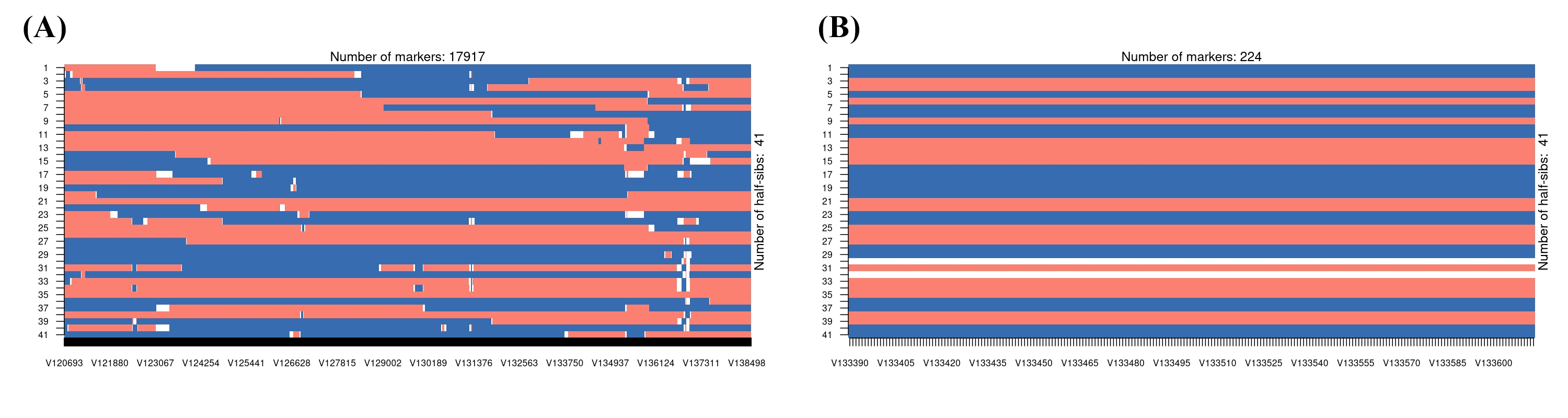
Supplementary Figure S18. Hsphase Imageplot of recombination events on BTA 7 (A) and on 82,974,837-83,997,563 interval (*Bos taurus* Genome assembly ARS-UCD1.2) (B) for sire’s 11 daughters.


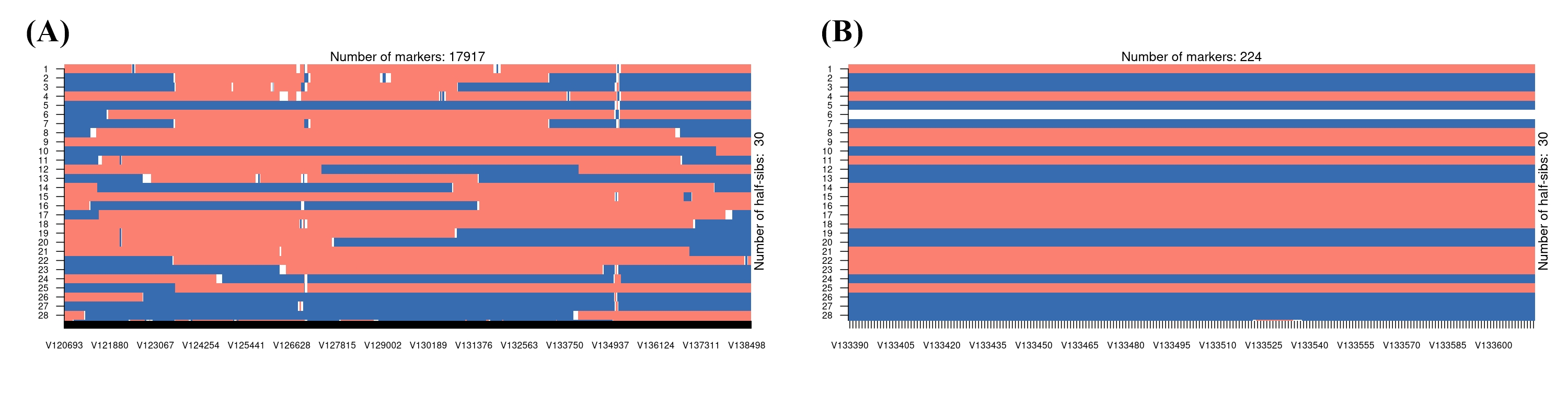
Supplementary Figure S19. Hsphase Imageplot of recombination events on BTA 7 (A) and on 82,974,837-83,997,563 interval (*Bos taurus* Genome assembly ARS-UCD1.2) (B) for sire’s 12 daughters.
